# Elucidating the Antiglycation Effect of Creatine on Methylglyoxal-Induced Carbonyl Stress In Vitro

**DOI:** 10.3390/ijms252010880

**Published:** 2024-10-10

**Authors:** Shin Koike, Haruka Mitsuhashi, Atsushi Kishida, Yuki Ogasawara

**Affiliations:** 1 Department of Analytical Biochemistry, Meiji Pharmaceutical University, 2-522-1 Noshio, Kiyose, Tokyo 204-8588, Japan; skoike@my-pharm.ac.jp (S.K.); y201286@std.my-pharm.ac.jp (H.M.); 2 Graduate School of Pharmaceutical Sciences, Meiji Pharmaceutical University, 2-522-1 Noshio, Kiyose, Tokyo 204-8588, Japan; akishida@my-pharm.ac.jp

**Keywords:** advanced glycation end products, antiglycation activity, carbonyl stress, creatine, methylglyoxal, oxidative stress

## Abstract

Advanced glycation end products (AGEs) with multiple structures are formed at the sites where carbonyl groups of reducing sugars bind to free amino groups of proteins through the Maillard reaction. In recent years, it has been highlighted that the accumulation of AGEs, which are generated when carbonyl compounds produced in the process of sugar metabolism react with proteins, is involved in various diseases. Creatine is a biocomponent that is homeostatically present throughout the body and is known to react nonenzymatically with α-dicarbonyl compounds. This study evaluated the antiglycation potential of creatine against methylglyoxal (MGO), a glucose metabolite that induces carbonyl stress with formation of AGEs in vitro. Further, to elucidate the mechanism of the cytoprotective action of creatine, its effect on the accumulation of carbonyl proteins in the cells and the MGO-induced cellular damage were investigated using neuroblastoma cells. The results revealed that creatine significantly inhibits protein carbonylation by directly reacting with MGO, and creatine added to the culture medium suppressed MGO-derived carbonylation of intracellular proteins and exerted a protective effect on MGO-induced cytotoxicity. These findings suggest that endogenous and supplemented creatine may contribute to the attenuation of carbonyl stress in vivo.

## 1. Introduction

Creatine is a naturally occurring nitrogen-containing compound synthesized from the amino acids glycine and methionine and classified within the guanidine phosphagen family [1,2]. While most research on creatine has focused on muscle, studies exploring its effects on the brain are gradually emerging. Creatine, derived from reactions involving the amino acids arginine, glycine, and methionine, is important for resynthesizing adenosine triphosphate, particularly during periods of increased metabolic demand [3,4,5]. Recently, creatine has become one of the most popular supplements for athletes; it can act as a cellular energy buffer and increase creatine phosphate and adenosine triphosphate regeneration [6]. In addition to their role as energy buffers, creatine compounds are known to have a variety of physiological effects. Creatine appears to exhibit significant antioxidant effects, which may result from various functional mechanisms, such as indirect mechanisms implicated in the cell membrane stabilization and improvement of cellular energy capacity [7] and its direct antioxidant properties [8].

In contrast, the role of creatine in carbonyl stress has been less studied. Löbner et al. [9] reported that ingested creatine may react with the glucose metabolite methylglyoxal (MGO), a carbonyl compound, forming N-(4-Methyl-5-oxo-1-imidazolin-2-yl) sarcosine (MG-HCr), which may subsequently remove MGO. They also reported that when MGO and creatine were simultaneously ingested in food, 56% of the MGO reacted with creatine, forming MGO-HCr in the human stomach [10]. Therefore, when ingested through the diet, MGO-HCr is considered a potential biomarker for the dietary intake of animal-source food [11]. However, the antiglycation potential of creatine has not been adequately studied, and the physiological role of creatine endogenously or ingested supplementary on carbonyl stress is unknown.

In living organisms, dicarbonyl compounds such as MGO, when present in excess, produce advanced glycation end products (AGEs), which in turn cause carbonyl stress. Pyridoxamine is well known as a direct scavenger of carbonyl compounds [12], and its activity has also been reported for guanidino compounds [13]. Therefore, we focused on the antiglycation effect of creatine, a biological component, and comprehensively examined its potential. This study evaluated the direct inhibitory effect of creatine on MGO-induced protein carbonylation. Moreover, whether creatine exerts its protective effect by inhibiting MGO-induced carbonylation in cultured neuronal cells was evaluated.

## 2. Results

### 2.1. Trapping of MGO by Creatine and the Formation of MGO-HCr

**Figure 1:**
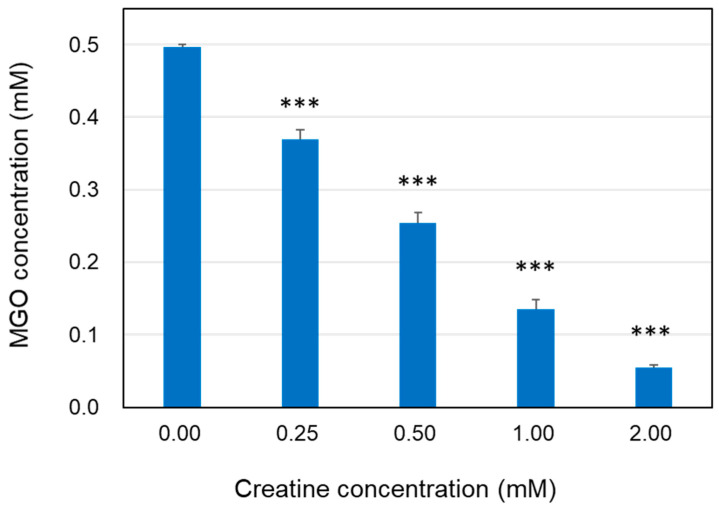
Decrease in MGO level in the presence of creatine. The reaction mixtures of MGO (0.5 mM) with creatine (0.25–2.0 mM) in PBS (pH 7.4) at 37 °C were incubated for 24 h. Methylglyoxal in the reaction mixture was quantified using HPLC-FL as described in the Section 4. Data are shown as the mean ± SD (*N* = 4). Statistical significance of differences was assessed using Dunnett’s multiple comparison test. *** *p* < 0.001 vs. MGO treatment without creatine (0 mM).

The reaction solution in which the free MGO disappeared was analyzed using HPLC-FL method. When MGO was incubated with various concentrations of creatine in PBS at 37 °C, the MGO concentration was clearly decreased after 24 h in a creatine concentration-dependent manner (Figure 1).

**Figure 2:**
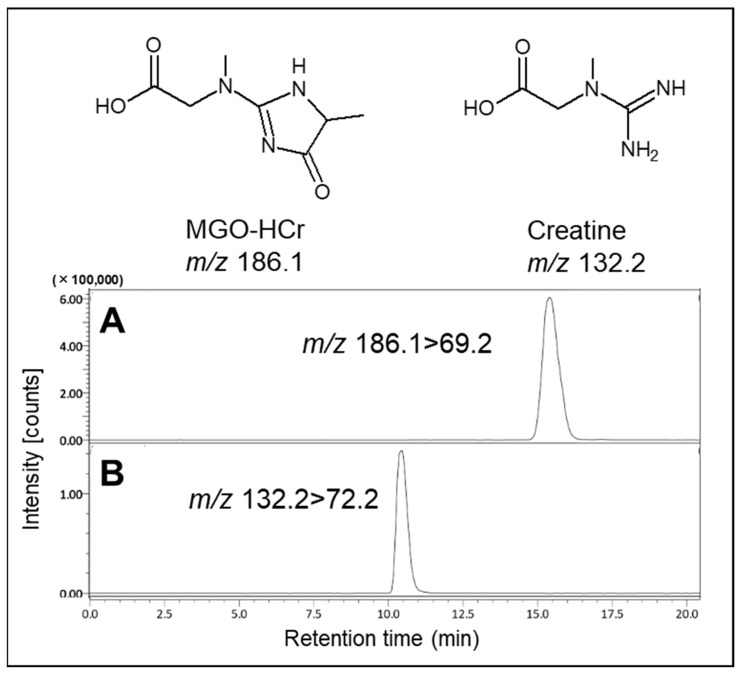
Identification of reaction product (MGO-HCr) in the presence of MGO and creatine. The tandem mass spectrometer was tuned in the multiple reaction monitoring (MRM) mode to monitor mass transitions in positive ion mode: (**A**) MGO-HCr; *m*/*z* 186.1–69.2, (**B**) creatine; *m*/*z* 132.2–72.2.

Based on this result, we attempted the formation of MGO-HCr, a previously reported Maillard reaction compound in foods [9]. In the present study, the formation of MGO-HCr was confirmed using LC-MS/MS in PBS containing 0.1 mM MGO and 0.1 mM creatine after incubation for 24 h at 37 °C (Figure 2). From these results, it was expected that creatine had the potential to trap MGO under physiological conditions through direct reaction.

### 2.2. Inhibitory Effects of Creatine on the Formation of MGO-Induced Fluorescent AGEs

**Figure 3:**
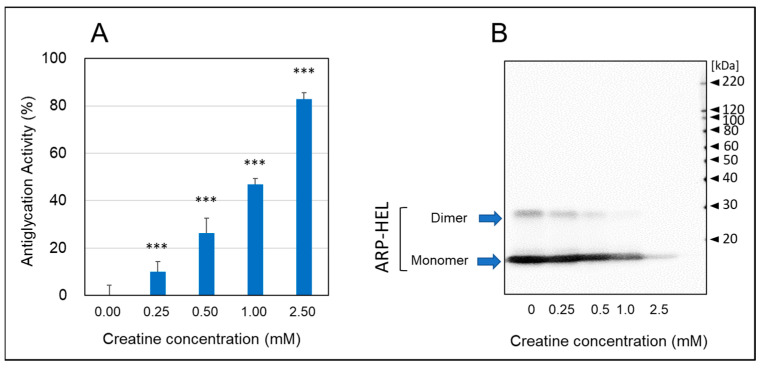
Anti-glycation activity by the HEL-MGO assay. Anti-glycation activities of various concentrations of creatine (0.25–2.0 mM) were assayed by HEL-MGO method. (**A**) The reaction mixtures of HEL with MGO (0.5 mM) and various concentrations of creatine (0.25–2.5 mM) in PBS (pH 7.4) at 37 °C were incubated for seven days. The inhibition rate (%) of the formation of ARP-modified HEL calculated by the decrease in fluorescent intensity compared to that of control (HEL + MGO) without treatment of creatine. Data are shown as the mean ± SD (*N* = 4). Statistical significance of differences was assessed using Dunnett’s multiple comparison test. *** *p* < 0.001 vs. MGO treatment without creatine (0 mM). (**B**) Western blotting analysis for ARP-modified HEL (monomer: 14.3 kDa and dimer 28 kDa) dependent on the creatine concentration.

As shown in Figure 3, when hen egg lysozyme (HEL) reacted with MGO and various concentrations of creatine for one week, the formation of argpyrimidine (ARP), a fluorescent AGE produced by the reaction with MGO, decreased in a creatine concentration-dependent manner (Figure 3A). Moreover, when the formation of ARP-modified HEL was detected by Western blot analysis, the inhibitory effect was clearly observed at additions of 0.5 mM creatine or higher, and ARP-modified HEL was barely detected with pretreatment of 2.0 mM creatine (Figure 3B). These results indicate that creatine inhibits MGO-induced protein carbonylation (ARP modification).

### 2.3. Inhibitory Effects of Creatine on the Steoric Conformational Change in BSA Induced by MGO

**Figure 4:**
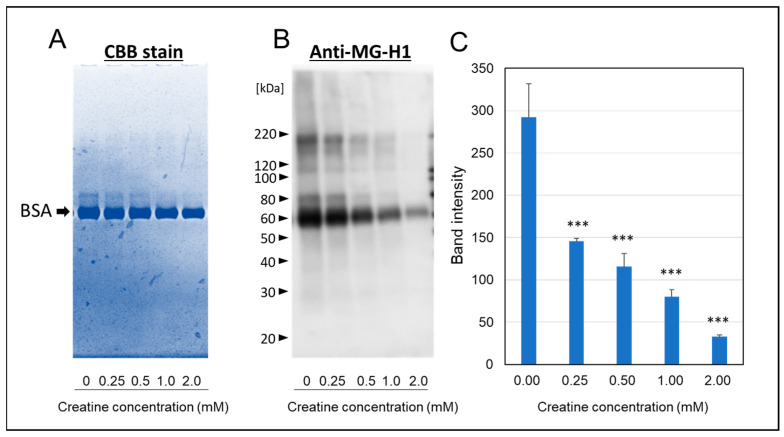
Effect of creatine on the MGO-induced stereoic conformational changes in BSA. The migration shift of modified BSA was detected by direct CBB staining (**A**) or Western blot analysis using anti-MG-H1 antibody (**B**) after one week of the carbonyl reaction of BSA with MGO and various concentrations of creatine (0.25–2.0 mM). The band intensities of MG-H1-modified BSA (as shown in (**B**) with or without creatine treatment were semi-quantitated by densitometry using Image Lab Version 5.2 (**C**). Data are shown as the mean ± SD (*N* = 3). Statistical significance of differences was assessed using Dunnett’s multiple comparison test. *** *p* < 0.001 vs. MGO treatment without creatine (0 mM).

As shown in Figure 4A, a portion of migration shift of modified-bovine serum albumin (BSA) (ca 80 kDa) was observed by SDS-PAGE with CBB staining. However, the simultaneous treatment of above 1.0 mM creatine completely inhibited the formation of modified-BSA shifted to polymeric side. Furthermore, we also found that MGO induced the steoric conformational change in BSA with calbonylation (MG-H1 modification) by the reaction at 37 °C for one week (Figure 4B). Also, the treatment of creatine with MGO significantly inhibited MGO-induced modification of BSA in a concentration-dependent manner (Figure 4C).

### 2.4. Inhibitory Effects of Creatine on the Formation of MG-H1-Modified Proteins in the SH-SY5Y Cells

**Figure 5:**
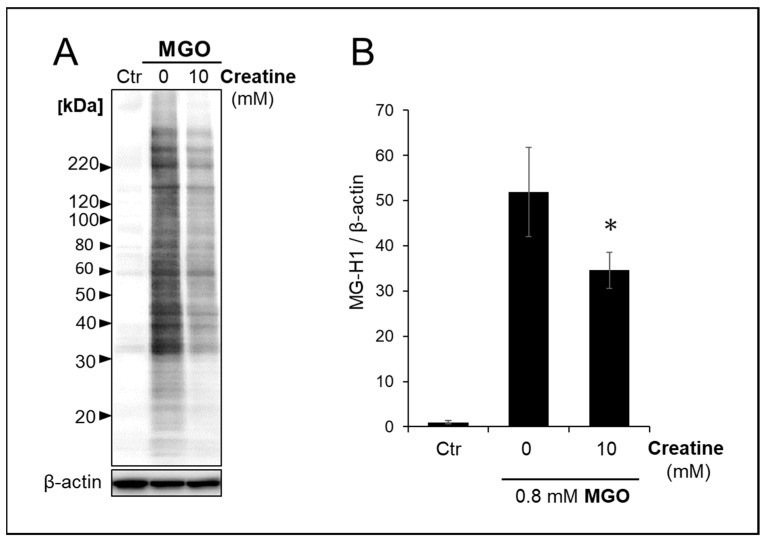
Effect of creatine on the accumulation of MG-H1-modified proteins treated with MGO. The vehicle (control) or 0.8 mM MGO with (10 mM) or without (0 mM) of creatine was added to the medium, and SH-SY5Y cells were cultured for 2 h. The MG-H1-modified proteins accumulated in the cells were detected by Western blot analysis with anti-MG-H1 antibody (**A**), and band intensities were semi-quantitated using densitometry software. (**B**). Data are shown as the mean ± SD (*N* = 3). Statistical significance of differences was assessed using Tukey’s multiple comparison test. * *p* < 0.05 vs. MGO treatment without creatine (0 mM).

We analyzed the effect of creatine on changes in carbonylated proteins produced and accumulated intracellularly in SH-SY5Y cells by MGO treatment. As shown in Figure 5A,B, the simultaneous addition of 10 mM creatine significantly reduced the accumulation of MGO-modified proteins bearing MG-H1 structures in the cells.

### 2.5. Neuroprotective Effects of Creatine as a Carbonyl Scavenger

**Figure 6:**
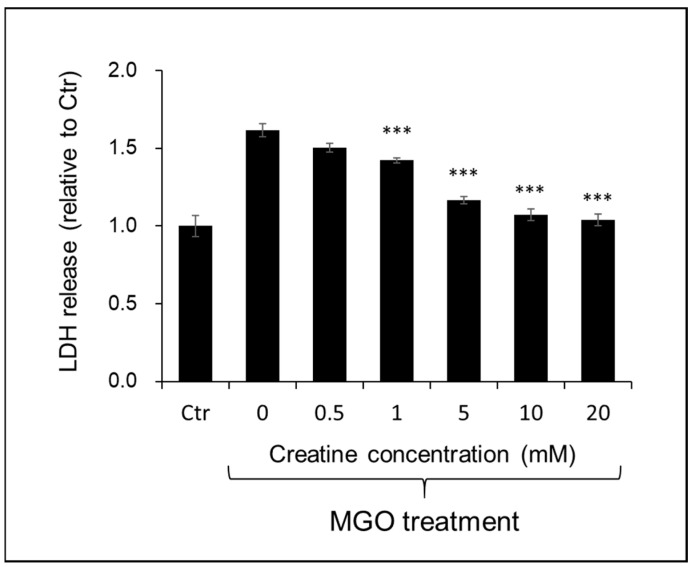
Effect of creatine on the MGO-induced cytotoxicity using SH-SY5Y cells. Cell viability was measured by LDH assay. Cells were treated with the vehicle (Ctr) or MGO (0.4 mM) along with creatine (0–20 mM) for 24 h. The LDH activity in the culture medium of each sample was measured at 490 nm. Data are shown as the mean ± SD (N = 4). Statistical significance of differences was assessed using Tukey’s multiple comparison test. *** *p* < 0.001 vs. MGO treatment without creatine (0 mM).

The effect of creatine on the cytotoxicity by MGO treatment to SH-SY5Y cells was examined by the LDH method. As shown in Figure 6, the simultaneous addition of creatine above 1 mM significantly attenuated MGO-induced cytotoxicity.

## 3. Discussion

There have been many reports of glycation and cytotoxicity caused by MGO [14], and AGEs induced by MGO have also been investigated in detail [15]. Furthermore, it has been reported that spontaneous reactions with MGO alter the structure and function of cellular macromolecules through the formation of inflammatory AGEs and the promotion of cytotoxic misfolding and the aggregation process [16,17,18,19]. Accumulation of MGO-induced AGEs in neural cells contributes to oxidative stress, a state of elevated inflammation commonly found in neurodegenerative diseases [20,21,22,23,24]. Recent research in food chemistry has extensively explored the ability of food ingredients and natural compounds to trap MGO [25,26,27]. However, few reports have evaluated the antiglycolytic activity of endogenous biocomponents against protein glycation by MGO, a glucose metabolite.

Creatine has been studied for its supplementation effects on systemic antioxidant capacity [28,29]. There are a few reports of direct antioxidant effects of creatine [6,30], but it is not considered to be a prominent antioxidant. The previous report [9] on the reaction of creatine with MGO in terms of antiglycation identified and characterized the reaction products by reacting high concentrations of MGO with high concentrations of creatine. Unfortunately, the physiological significance of this reaction remains unclear, and its product, MGO-HCr, is thought to be present in gastrointestinal digests and foods. Subsequently, we cannot find any research reports on the reaction of creatine as a biocomponent with dicarbonyl compounds. Thus, its antiglycation activity has not been well studied, and its inhibitory effect on carbonylation in the cell remains unknown. This study focused on the inhibitory effect of creatine, which is abundant in the brain, on MGO-induced carbonylation and evaluated it in detail. The results of this study suggest that creatine directly reacts with MGO to form MGO-HCr, which inhibits MGO-induced ARP formation in HEL, as well as steric conformational changes and MG-H1 formation in BSA. These results indicated that creatine has the potential to suppress protein carbonylation in vitro. Furthermore, in the present study using the human-derived SH-SY5Y cell, it was shown for the first time that simultaneous treatment with creatine reduced the toxicity of MGO and inhibited the accumulation of MG-H1-modified proteins, one of the MGO-induced AGEs generated in the cells. This may be because MGO added to the medium reacted with creatine to produce MGO-HCr, which in turn prevented MGO from reacting with proteins in the SH-SY5Y cells. Thus, the results of this study suggest that endogenous and supplemented creatine may react with excessively generated MGO to reduce the carbonylation of proteins, thereby decreasing their cytotoxicity.

There have been many reports that daily creatine supplementation is beneficial in the prevention of neurodegenerative diseases, including Alzheimer’s disease [31,32,33]. Although most of those experiments have been conducted in terms of improving energy metabolism and maintaining and improving mitochondrial function by creatine with ATP production in the brain, the detailed mechanisms are still unclear. In the future, it will be required to conduct in vivo experiments using model animals, focusing on the anti-glycation and anti-oxidation effects of creatine. Furthermore, considering the conditions of many preclinical studies that have been performed, creatine administration at approximately 10–100 mg/kg per day (p.o.) or a diet containing 2–3% creatine per day would be expected to clarify the effect of creatine on the prevention of neurodegenerative diseases.

## 4. Materials and Methods

### 4.1. Materials

Acetonitrile and formate were of LC-MS grade (Thermo Scientific Chemicals, Waltham, MA, USA). All aqueous solutions were prepared with pure water produced by Milli-Q system (Merck-Millipore, Tokyo, Japan). BSA, HEL, and 1,2-diamino-4,5-methylenedioxybenzene (MDB) were purchased from Fujifilm-Wako Chemical (Osaka, Japan). CBB staining solution was from Apro Science (Tokushima, Japan). Purified MGO was prepared by the method previously reported [34].

### 4.2. Estimation of MGO-Trapping Ability by HPLC Method

The ability of creatine to trap MGO was determined according to residual amounts of free MGO. The reaction mixtures of MGO (0.5 mM) with creatine (0.25–2.0 mM) in PBS (pH 7.4) at 37 °C were incubated for 24 h. MGO in the reaction mixture was quantified by prelabeling method using MDB and high-performance liquid chromatography with fluorescence detection (HPLC-FL), as described previously [35,36].

### 4.3. Identification of MGO-Derived Product with Creatine by LC–MS/MS Analysis

Analysis of MGO-HCr was determined by LC-MS/MS method according to the previous report [9] with modification.

The LC–MS/MS system consisted of LC-30AD pumps, a SIL-30AC auto-sampler, a CTO-20A column oven, a CBM-20A system controller (Shimadzu, Kyoto, Japan), and a triple quadrupole mass spectrometer LCMS-8040 (Shimadzu, Kyoto, Japan). Samples were separated using a CAPCELL PAK ADME-HR column (5 µm, 4.6 mm i.d. × 150 mm; OSAKA SODA Co., Ltd., Osaka, Japan).

Elution was performed at 40 °C with a flow rate of 0.2 mL/min with 0.1% formic acid in water. The effluent was subjected to mass spectrometry using an electrospray ionization (ESI) interface operating in the positive-ion mode. The source temperature was set at 400 °C, and the ion spray voltage was 4.5 kV. Nitrogen was used as a nebulizer and drying gas. The tandem mass spectrometer was tuned in the multiple reaction monitoring mode to monitor the mass transitions *m/z* Q1/Q3 132.2/72.2 (creatine) and 186.1/69.2 (MGO-HCr).

### 4.4. Hen Egg Lysozyme (HEL)-MGO Assay with Fluorometric Detection and Western Blot Analysis

According to the previous study [37], HEL (1.0 mg/mL) with MGO (0.5 mM) and various concentrations of creatine (0.25–2.5 mM) were incubated at 37 °C in PBS for seven days. After appropriate dilution by PBS, the fluorescence was immediately measured at 390 nm, exciting at 320 nm, using a fluorospectrometric microplate reader (Perkin Elmer, EnSpire, South Plainfield, NJ, USA). Antiglycation activity was expressed as the percentage of inhibition on the formation of fluorescent AGEs [17]. Also, antiglycation activity was evaluated from the decrease in band intensities of ARP-modified HEL (monomer and dimer) by Western blot analysis using anti-ARP antibody.

### 4.5. Assay for Steric Conformational Change in MGO-Modified BSA Using SDS-PAGE and Western Blot Analysis

BSA (1.0 mg/mL) was incubated with MGO (0.5 mM) and various concentrations of creatine (0.25–2.0 mM) at 37 °C in PBS for seven days. Modification of BSA induced by MGO and the inhibitory effect of creatine against the modification was detected by SDS-PAGE analysis followed by Coomassie Brilliant Blue (CBB) staining or Western blot analysis using anti-MG-H1 antibody.

### 4.6. Cell Culture

The human neuroblastoma cell line SH-SY5Y (ATCC No.: CRL-2266) has been used by many groups for toxicity experiments related to Alzheimer’s disease. Cell culture was performed as described previously [38,39]. Briefly, the cells were seeded into 96-well flat bottom tissue culture plates (100 μL vol.) at a density of 3 × 10^4^ cells /cm^2^. Cells were grown for 48 h in DMEM/F12 supplemented with 10% fetal bovine serum (FBS), penicillin/streptomycin (100 U/mL, 100 μg/mL) and 2 mM L-glutamine at 37 °C in a humidified atmosphere containing 5% CO_2_/95% air. After 48 h, cells were washed with fresh medium. MGO, along with creatine, were added in the culture medium after replacement with fresh medium.

### 4.7. Cell Treatment with MGO and Creatine on the Accumulation of MG-H1-Modified Protein

SH-SY5Y cells were co-treated with MGO and creatine. Briefly, cells were seeded at a density of 2 × 10^4^ cells/cm^2^ into a culture dish with DMEM/F12 medium containing 10% FBS and incubated for 48 h. The culture medium was replaced with fresh medium containing 2% FBS before MGO (0.8 mM) treatment of cells with (10 mM) or without (0 mM) creatine.

### 4.8. Cell Survival Assay

SH-SY5Y cells were seeded onto a 96-well plate at a density of 2 × 10^4^ cells/cm^2^ in 10% FBS containing DMEM/F12 medium. The medium was changed to the DMEM/F12 medium containing 2% FBS, followed by co-treatment with 0.4 mM MGO and various concentrations of creatine. Moreover, 24 h later, LDH leakage was measured using LDH Cytotoxicity Detection Kit (TAKARA BIO Inc., Shiga, Japan) according to the manufacturer’s instructions. The absorbance of the sample was measured at 490 nm with a microplate reader (PerkinElmer EnSpire, Waltham, MA, USA).

### 4.9. Western Blot Analysis

Samples were heated for 5 min at 90 °C in SDS sample buffer with a reducing reagent (Nacalai Tesque, Kyoto, Japan). The proteins were separated by SDS–polyacrylamide gel electrophoresis (5–20% gradient gel, ATTO, Tokyo, Japan) and transferred to polyvinylidene difluoride (PVDF) membranes (Millipore, Billerica, MA, USA). Non-specific binding to the membrane was blocked by using 4% (*w*/*v*) Block Ace (DS Pharma Biomedical, Osaka, Japan). The washed PVDF membranes were then incubated for 1 h at room temperature with a primary, anti-MG-H1 (Cell Biolabs, San Diego, CA, USA), anti-albumin (Bioss Inc., Woburn, MA, USA), anti-ARP (monoclonal, NIKKEN SEIL Co., Ltd., Shizuoka, Japan), β-actin conjugated with HRP (Cell Signaling Technology, Danvers, MA, USA). After extensive washing in TPBS, the blots were incubated at room temperature for 1 h with anti-mouse IgG horseradish peroxidase-conjugated secondary antibodies (Vector, Burlingame, CA, USA). The bands were imaged using chemiluminescence reagents (Crescendo; Merck Millipore, Darmstadt, Germany) with a ChemiDoc^TM^ Touch Imaging System (Bio-Rad, Hercules, CA, USA). The band intensities in the images were quantified by Image Lab™ Version 5.2 (Bio-Rad, Laboratories, Tokyo, Japan).

### 4.10. Statistical Analysis

Values are presented as the mean ± standard deviation (SD) of at least three independent experiments. Statistical significance of differences was assessed using Dunnett’s multiple comparison test or Tukey’s multiple comparison test (*p* < 0.05 was considered to be a statistically significant difference).

## Data Availability

The data are contained within the article.
